# Supplementary material for: End Sequence Analysis Toolkit (ESAT) expands the extractable information from single-cell RNA-seq data
Source: Genome Res. 2016 Oct;26(10):1397–410. doi: 10.1101/gr.207902.116 (PMC5052061; doi:10.1101/gr.207902.116)
Supplement: Supplemental Material [file supp_gr.207902.116_Supp_Materials.tar.gz › ESAT-esat_v0.1/doc/html/classbroad_1_1pda_1_1seq_1_1fastq_1_1_fastq_splitter_by_barcode.html]

ESAT: broad.pda.seq.fastq.FastqSplitterByBarcode Class Reference


ESAT


- Main Page
- Related Pages
- Packages
- Classes
- Files

- Class List
- Class Index
- Class Hierarchy
- Class Members

All Classes Namespaces Files Functions Variables Enumerator Pages

- broad
- pda
- seq
- fastq
- FastqSplitterByBarcode

Public Member Functions |
Static Public Member Functions |
Protected Member Functions |
List of all members

broad.pda.seq.fastq.FastqSplitterByBarcode Class Reference

Collaboration diagram for broad.pda.seq.fastq.FastqSplitterByBarcode:

[legend]

|  |  |
| --- | --- |
| Public Member Functions | |
|  | FastqSplitterByBarcode (String bcFile) throws IOException |
|  | |
| void | stop () throws IOException |
|  | |
| void | start (String pair1Source, String outputDir, String pair2Source) throws IOException |
|  | |
| boolean | hasMore () |
|  | |
| boolean | isPaired () |
|  | |
| void | setPaired (boolean paired) |
|  | |
| boolean | isAtEnd () |
|  | |
| void | setAtEnd (boolean atEnd) |
|  | |
| int | getRead2Trim5p () |
|  | |
| void | setRead2Trim5p (int read2Trim5p) |
|  | |
| int | getRead2Trim3p () |
|  | |
| void | setRead2Trim3p (int read2Trim3p) |
|  | |
| int | getRead1Trim3p () |
|  | |
| void | setRead1Trim3p (int read1Trim3p) |
|  | |
| int | getRead1Trim5p () |
|  | |
| void | setRead1Trim5p (int read1Trim5p) |
|  | |
| boolean | isAddToReadName () |
|  | |
| void | setAddToReadName (boolean addToReadName) |
|  | |

|  |  |
| --- | --- |
| Static Public Member Functions | |
| static void | main (String[] args) throws Exception |
|  | |

|  |  |
| --- | --- |
| Protected Member Functions | |
| void | updateReadInfoWithBarcode (FastqSequence read, String bc) |
|  | |
| FastqParser | getPair1Parser () |
|  | |
| void | setPair1Parser (FastqParser pair1Parser) |
|  | |
| FastqParser | getPair2Parser () |
|  | |
| void | setPair2Parser (FastqParser pair2Parser) |
|  | |

## Detailed Description

Reads through a fastq file and writes records to different files depending on the barcode found within the start or end of the read. Reads are trimmed to remove the barcode.

Author
:   mgarber

## Constructor & Destructor Documentation

|  |  |  |  |  |  |
| --- | --- | --- | --- | --- | --- |
| broad.pda.seq.fastq.FastqSplitterByBarcode.FastqSplitterByBarcode | ( | String | *bcFile* | ) | throws IOException |

Here is the caller graph for this function:

## Member Function Documentation

|  |  |  |  |  |
| --- | --- | --- | --- | --- |
| FastqParser broad.pda.seq.fastq.FastqSplitterByBarcode.getPair1Parser | ( |  | ) |  |

protected

|  |  |  |  |  |
| --- | --- | --- | --- | --- |
| FastqParser broad.pda.seq.fastq.FastqSplitterByBarcode.getPair2Parser | ( |  | ) |  |

protected

|  |  |  |  |  |
| --- | --- | --- | --- | --- |
| int broad.pda.seq.fastq.FastqSplitterByBarcode.getRead1Trim3p | ( |  | ) |  |

|  |  |  |  |  |
| --- | --- | --- | --- | --- |
| int broad.pda.seq.fastq.FastqSplitterByBarcode.getRead1Trim5p | ( |  | ) |  |

|  |  |  |  |  |
| --- | --- | --- | --- | --- |
| int broad.pda.seq.fastq.FastqSplitterByBarcode.getRead2Trim3p | ( |  | ) |  |

|  |  |  |  |  |
| --- | --- | --- | --- | --- |
| int broad.pda.seq.fastq.FastqSplitterByBarcode.getRead2Trim5p | ( |  | ) |  |

|  |  |  |  |  |
| --- | --- | --- | --- | --- |
| boolean broad.pda.seq.fastq.FastqSplitterByBarcode.hasMore | ( |  | ) |  |

Here is the caller graph for this function:

|  |  |  |  |  |
| --- | --- | --- | --- | --- |
| boolean broad.pda.seq.fastq.FastqSplitterByBarcode.isAddToReadName | ( |  | ) |  |

|  |  |  |  |  |
| --- | --- | --- | --- | --- |
| boolean broad.pda.seq.fastq.FastqSplitterByBarcode.isAtEnd | ( |  | ) |  |

|  |  |  |  |  |
| --- | --- | --- | --- | --- |
| boolean broad.pda.seq.fastq.FastqSplitterByBarcode.isPaired | ( |  | ) |  |

Here is the caller graph for this function:

|  |  |  |  |  |  |
| --- | --- | --- | --- | --- | --- |
| static void broad.pda.seq.fastq.FastqSplitterByBarcode.main | ( | String[] | *args* | ) | throws Exception |

static

Here is the call graph for this function:

|  |  |  |  |  |  |
| --- | --- | --- | --- | --- | --- |
| void broad.pda.seq.fastq.FastqSplitterByBarcode.setAddToReadName | ( | boolean | *addToReadName* | ) |  |

|  |  |  |  |  |  |
| --- | --- | --- | --- | --- | --- |
| void broad.pda.seq.fastq.FastqSplitterByBarcode.setAtEnd | ( | boolean | *atEnd* | ) |  |

|  |  |  |  |  |  |
| --- | --- | --- | --- | --- | --- |
| void broad.pda.seq.fastq.FastqSplitterByBarcode.setPair1Parser | ( | FastqParser | *pair1Parser* | ) |  |

protected

|  |  |  |  |  |  |
| --- | --- | --- | --- | --- | --- |
| void broad.pda.seq.fastq.FastqSplitterByBarcode.setPair2Parser | ( | FastqParser | *pair2Parser* | ) |  |

protected

|  |  |  |  |  |  |
| --- | --- | --- | --- | --- | --- |
| void broad.pda.seq.fastq.FastqSplitterByBarcode.setPaired | ( | boolean | *paired* | ) |  |

|  |  |  |  |  |  |
| --- | --- | --- | --- | --- | --- |
| void broad.pda.seq.fastq.FastqSplitterByBarcode.setRead1Trim3p | ( | int | *read1Trim3p* | ) |  |

|  |  |  |  |  |  |
| --- | --- | --- | --- | --- | --- |
| void broad.pda.seq.fastq.FastqSplitterByBarcode.setRead1Trim5p | ( | int | *read1Trim5p* | ) |  |

|  |  |  |  |  |  |
| --- | --- | --- | --- | --- | --- |
| void broad.pda.seq.fastq.FastqSplitterByBarcode.setRead2Trim3p | ( | int | *read2Trim3p* | ) |  |

|  |  |  |  |  |  |
| --- | --- | --- | --- | --- | --- |
| void broad.pda.seq.fastq.FastqSplitterByBarcode.setRead2Trim5p | ( | int | *read2Trim5p* | ) |  |

|  |  |  |  |
| --- | --- | --- | --- |
| void broad.pda.seq.fastq.FastqSplitterByBarcode.start | ( | String | *pair1Source*, |
|  |  | String | *outputDir*, |
|  |  | String | *pair2Source* |
|  | ) |  | throws IOException |

Here is the call graph for this function:

|  |  |  |  |  |
| --- | --- | --- | --- | --- |
| void broad.pda.seq.fastq.FastqSplitterByBarcode.stop | ( |  | ) | throws IOException |

Here is the call graph for this function:

|  |  |  |  |
| --- | --- | --- | --- |
| void broad.pda.seq.fastq.FastqSplitterByBarcode.updateReadInfoWithBarcode | ( | FastqSequence | *read*, |
|  |  | String | *bc* |
|  | ) |  |  |

protected

Here is the call graph for this function:

---

The documentation for this class was generated from the following file:

- java/broad/pda/seq/fastq/FastqSplitterByBarcode.java


---

Generated on Fri Aug 15 2014 15:11:02 for ESAT by  

 1.8.7
